# Family Dog-Assisted Adapted Physical Activity: A Case Study

**DOI:** 10.3390/ani7050035

**Published:** 2017-04-27

**Authors:** Amanda Tepfer, Samantha Ross, Megan MacDonald, Monique A. R. Udell, Craig Ruaux, Wendy Baltzer

**Affiliations:** 1 Health & Human Performance, Norwich University, Northfield, VT 05663, USA; atepfer@norwich.edu; 2 College of Public Health & Human Sciences, Oregon State University, Corvallis, OR 97331, USA; Samanatha.Ross@oregonstate.edu; 3 Department of Animal & Rangeland Sciences, Oregon State University, Corvallis, OR 97331, USA; monique.udell@oregonstate.edu; 4 College of Veterinary Medicine, Oregon State University, Corvallis, OR 97331, USA; Craig.Ruaux@oregonstate.edu; 5 Institute of Veterinary, Animal and Biomedical Sciences, Massey University, Palmerston North 4442, New Zealand; w.baltzer@massey.ac.nz

**Keywords:** cerebral palsy, adapted physical activity, animal assisted intervention, CPQOL-Child, motor skills

## Abstract

**Simple Summary:**

Understanding how family dogs aid in aspects of daily living such as quality of life, physical activity and human animal interaction is critical towards better understanding child health. Using questionnaires and direct assessment we aimed to better understand the role of the family dog in an animal assisted adapted physical activity intervention. Findings were positive in respect to all primary outcomes in this case study. Generally, the role of the family dog in an adapted physical activity animal assisted intervention had positive results for child health, when the family dog assisted a child with cerebral palsy in this type of intervention.

**Abstract:**

*Purpose:* The aim of this case study was to examine the individual effects of an adapted physical activity, animal-assisted intervention (APA-AAI) with the family dog on motor skills, physical activity, and quality of life of a child with cerebral palsy (CP). *Method*: This study used an A-B-A single-subject design. The assessment phase (phase A) occurred pre- and post-intervention. This consisted of standardized assessments of motor skills, quality of life questionnaires, physical activity (measured using the GT3X+ accelerometer) and the human-animal bond. The intervention (phase B) lasted 8 weeks and consisted of adapted physical activities performed with the family dog once a week for 60 min in a lab setting. In addition, the participant had at-home daily activities to complete with the family dog. *Results:* Visual analysis was used to analyze the data. Motor skill performance, physical activity, quality of life and human animal interaction gains were observed in each case. *Conclusions:* These preliminary results provided initial evidence that the family-dog can play a role in healthy lifestyles through APA-AAI in children with CP.

## 1. Introduction 

Children with cerebral palsy (CP) spend significantly less time participating in physical activity compared to typically developing peers [1,2,3]. Children with CP demonstrate delays in motor skill development, coordination and balance resulting in physical activity (PA) limitations [4]. Furthermore, research has indicated that children with CP are physically weaker, less fit, and have lower cardiovascular endurance compared to typically developing children [5,6,7,8,9]. This places children with CP at greater risk for developing secondary conditions associated with physical inactivity, such as chronic pain, fatigue and osteoporosis. Participation in regular PA is associated with several important health benefits including improved body composition, musculoskeletal health, cardiorespiratory fitness, and increased self-concept and self-esteem [10,11]. For children with CP, activity limitations, associated with this disorder, may present unique PA participation barriers. Therefore, the development of effective PA promotion programs is critical to ensure optimal health outcomes for this at risk group. 

Traditional therapy programs for children with CP (e.g., physical or occupational therapy) have not demonstrated consistent effects on increased activity nor have they reported better or increased gains over alternative methods, such as horseback riding or hydrotherapy [12,13]. Furthermore, children often regress following traditional therapy programs when the program ends, as long-term adherence to therapy protocols remains low [14,15]. Creating unique physical activity opportunities, with potential long-term effects for children with disabilities, is critical to improving overall health. One emerging physical activity option is animal assisted therapy (AAT), a goal-directed intervention with the inclusion of an animal [16,17]. Animal-assisted PA interventions, using certified therapy dogs, have been shown to improve PA [18], motor skills and performance related tasks [19,20,21] in typically developing children. Furthermore, the presence of personal service dogs have been associated with improved psychological well-being and self-reported quality of life among adults with mobility disabilities [22]. To our knowledge, there are no known studies that have examined the motor skills, quality of life benefits, and human animal interactions of animal-assisted interventions using the family dog within a physical activity program for children with CP. 

Many AAIs are limited to trained service or therapy dogs, which may only be present during the intervention period (e.g., one dog may be serving multiple children in a specific program). The inclusion of the family dog may allow the dog to play a more full-time role in the intervention and provide support, motivation and companionship within and beyond the child’s home [23]. Family dog-ownership has been positively associated with average daily step-counts in typically developing children [24,25]. Among a cohort of children 10–12 years, children with a family dog were 49% more likely to achieve the recommended amount of PA per week compared to children who were non family dog owners [26].

Incorporating the family dog in AAI programs has the potential to more actively promote daily physical activity among children with disabilities, a group at risk for low participation. However, little is presently known about the effects of a physical activity AAI for children with CP. Therefore, the purpose of this study was to examine the individual effects of a physical activity based AAI with the family dog on the motor skills, physical activity, and quality of life of one child with CP. The effects of these activities on the child-dog bond were also evaluated. Based on previous research, it was hypothesized that participation with the family dog in an individualized physical activity program would be associated with gains in motor skills, physical activity, quality of life and improved human animal interactions.

## 2. Methods

### 2.1. Participants

Child: The participant in this report is a 10-year-old male. Child 1 was diagnosed with CP, per parental report. CP was confirmed using the Gross Motor Function Classification System—Expanded and Revised (GMFCS-E&R) [27] and was indicated at level 2 (walks with some assistance). There were no other health conditions reported by the parent.

Dog: We partnered the child participant with a one-year old male Pomeranian who was already owned by the participant’s family. Prior to the intervention this dog had no formal training history and had been living indoors as a pet. This was the only dog currently living in the household and had been living in the household since 8-weeks of age. The dog was evaluated by a veterinarian and found to be healthy prior to inclusion in the intervention.

### 2.2. Study Design

A single-subject A-B-A design, consisting of a baseline assessment (A), intervention (B), post-intervention assessment (A). The baseline phase consisted of consent, initial motor skill, physical activity, quality of life and human animal interaction assessments. The intervention phase consisted of an 8-week intervention held at a veterinary clinic once per week for 60 min and daily at-home activities assigned by the interventionist (see Table 1). Post-intervention follow-up occurred eight weeks (immediately following the intervention), where all assessments were administered. 

### 2.3. Assessments 

**Gross Motor Function Classification System-Expanded and Revised (GMFCS-E&R)**. The GMFCS-E&R is used to classify children with CP based on age, functional and ambulatory ability. The GMFCS-E&R includes five age bands (under 2 years, between 2–4 years, between 4–6 years, between 6–12 years, and between 12–18 years) and five-levels (level I represents the least activity limitations and level V the most) [28]. This version (expanded and revised) includes concepts from the International Classification of Functioning, Disability, and Health (World Health Organization (WHO), [29]) noting gross motor function is influenced by physical, social, and attitudinal environments as well as personal factors such as preferences, interests, and motivation [28]. The GMFCS-E&R demonstrates content validity, intra- and inter-rater reliability and has been reported to remain stable over time [30,31,32]. In this study, the GMFCS-E&R was used to confirm a diagnosis of CP.

**Test of Gross Motor Development, 2nd Edition (TGMD-2)**. The TGMD-2 is a standardized test designed to assess the gross motor skills of children aged 3 through 10 years [33]. The TGMD-2 consists of 12 fundamental motor skills, and the process of each motor skill is assessed (e.g., multiple components of the skills are assessed, not just the final outcome of the skill). These skills include 6 locomotor skills; running, hopping, galloping, jumping, leaping and sliding and 6 object control skills (ball skills); throwing, striking, catching, dribbling, kicking, and rolling). The TGMD-2 demonstrates high test-retest reliability as well as content, criterion-prediction, and construct-identification validity in typically developing children [34]. 

**CP Quality of Life Questionnaire for Children (CPQOL-Child)**. The CP QOL-Child is a condition-specific quality of life (QOL) questionnaire for children between the ages of 4–12 years with CP [33]. The CPQOL-Child consists of two questionnaires: a child reported questionnaire (for children 9–12 years) and a primary caregiver reported questionnaire (4–12 years) that the participant’s parent/caregiver completed. Questions on these questionnaires are aimed at measuring quality of life of children with CP across five domains: social well-being and acceptance, participation and physical health, feelings about functioning, emotional well-being and self-esteem, pain and impact of disability. The CPQOL-Primary Caregiver measures an additional domain, access to services and family health. These questionnaires have demonstrated a high level of internal consistency and test-retest reliability and take approximately 20–30 min to complete [35].

**Physical Activity**. The Actigraph GT3X+ (Pensacola, FL, USA) was used to measure physical activity. The Actigraph GT3X+ is a small, lightweight tri-axial accelerometer, which measures and records accelerations in the vertical, horizontal and perpendicular axis. The GT3X+ was set to collect acceleration data at 30 Hz (30 data points per second), which is recommended for capturing human activity [36]. The participant was instructed to wear the device on the right hip via an elastic band for seven consecutive days and given a log to track when the accelerometer was worn. The participant was told he should take off the accelerometer for swimming, bathing or sleeping. This accelerometer protocol is similar to other studies measuring physical activity [37]. 

ActiLife software, version 6.8 (ActiGraph Corp., Pensacola, FL, USA), was used to process the initial, unfiltered data. Once data were downloaded, the ActiLife software extracted data of the vertical axis in 30-s epochs. PA intensity was divided by counts per minute (CPM) into the categories of sedentary (0–100 cpm), light (101–2295 cpm), and moderate-to-vigorous physical activity (MVPA) (2296 cpm and above) [38]. These cut point values were chosen based on MVPA interpretation for children and adolescents [37]. PA data was analyzed if the participant engaged in a minimum of 2 days of at least 9 h per day of valid accelerometer recordings [39]. Non-wear time, defined as any consecutive string of min with zero counts of at least 20 min, was excluded from the analysis. 

#### Human Animal Interaction Assessments

**Attachment Test**. The exploratory, avoidance, and social behavior of the dog was assessed in a two-minute reunion session that followed a habituation (2 min) and alone (2 min) phase. The dog was tested in an examination room located in the small animal hospital of Oregon State University’s veterinary medicine teaching clinic. There was a small desk and two chairs located in the room. For the attachment test three toys were also present. The setup with the same for all phases of this test as well as for pre and post intervention assessments. During habituation the child sat in a chair and was told only to acknowledge the dog with petting if it entered a 1 m-radius circle. After this period the child was directed to stand up and leave the room, leaving the dog alone inside. The child was then instructed to reenter the room, and behave in the same manner as during the habituation phase. The reunion upon the child’s return, and the two min that followed were video recorded and coded by two independent coders. The two-minute period was divided into 10-s bins; the coder recorded if any of the following behaviors occurred within each time bin based on standard attachment categorizations [40]:

**Contact-seeking behaviors**—Approaching within 1 m, any contact, including touching, leaning, nuzzling, licking or jumping on child, or play with child.
**Exploratory**—Exploring the room or independent play.
**Avoidance**—Sitting, standing, or laying away from child contact.

In the case of a secure attachment it was expected that the dog would engage in high levels of contact-seeking behavior in the first minute upon the child’s return, and that this behavior would either decrease in the second minute and/or be accompanied by exploratory behavior. Absence of contact seeking behavior (avoidance) during the first minute is associated with an insecure-avoidant attachment, and prolonged contact seeking behavior that extends into the second minute in the absence of exploratory behavior has been associated with an insecure-ambivalent (or dependent) attachment [40]. 

**Sociability**. The dog’s sociability towards the child was also assessed pre- and post-therapy in the same room, except toys were removed prior to this test. This consisted of a two minute passive phase (where the child sat quietly in a chair and was instructed not to talk to or interact with the dog unless it came within reaching distance, at which time they could pet it) and a two minute active phase (where the child was instructed to actively call the dog, trying to gain and maintain contact without leaving the chair, but also without physically restraining the dog). The duration of time the dog spent in proximity to (1 m) to the child was recorded from video.

**Gesture Responsiveness**. This test was used to assess how responsive the dog was to the non-verbal actions of the child prior to and after the intervention. Gesture responsiveness is frequently used as a measure of the human-dog bond; additionally, gesture responsiveness can facilitate positive interactions and cross-species communication, which are especially important in training and working contexts [41]. The child sat in a chair between two equidistant choice containers located 1 m in front and to the left and right of their centre line. An assistant held the dog behind a start line located 2.5 m away from the centre line between the containers. Food treats could be placed within each container so that it was free (providing a reward for correct choices), or trapped (for incorrect choices), allowing for equal smell cues and while looking identical from the dog’s point of view. Prior to testing food was made available on top of each container twice, alternating locations, and the dog was allowed to freely consume it. This was done to create an association between the containers and the availability of food. After this, dogs were given 10 trials to locate the accessible hidden food in one of the two locations. On each trial, the child was instructed to point to the correct container, as indicated by the experimenter, using their ipsilateral arm and hand (while remaining in the chair) until their dog made a choice. The correct container was determined pseudorandomly before sessions by the experimenter, subject to the constraints that no one location was designated correct more than twice in a row and each location was correct for 50% of the trials. If the dog approached the correct (pointed to) container first it was allowed to uncover and eat the treat found under the container. If the dog approached the incorrect container first, it received no food reward and was led back to the starting line to begin the next trial. If the dog failed to approach either container within 30 s it was treated as an incorrect response, and the dog was brought back to start the next trial.

### 2.4. Intervention

The AAI was designed to promote physical activity and gross motor function in children with CP with their family dog. The activities implemented were intended to be user friendly, cost efficient and easy to complete in a variety of environments (e.g., outside or inside the home). The intervention consisted of two parts. Part 1 consisted of a 60-min session, once per week, for 8 weeks at the veterinary clinic with a trained research assistant and certified canine rehabilitation practitioner. Each intervention was tailored to the participant and followed or modified (as needed) the content listed in Table 1.

Part 2 of the physical activity AAI consisted of the participant performing daily activities at home (6-days per week (the days the intervention did not take place)), with the family dog. The participant was given an activity log to record the amount of time or number of repetitions for each activity. Activities were similar to intervention activities for consistency, but did not include cavelettis or balance board/disc activities. In lieu of the omitted activities the participant was asked to log how long they walked with their dog and/or family.

### 2.5. Procedures

The Institutional Review Board (IRB) and Institutional Animal Care and Use Committee (IACUC) approved all methods and procedures for this study (IRB, 7049; ACUC, 4444). Parents and participants provided written consent and assent prior to any study procedures. The first baseline phase consisted of initial assessments in motor function, motor skills, physical activity, quality of life and human animal interactions.

Upon completing the 8-week intervention each participant was re-assessed with the same initial baseline measures. Post-follow up assessments were conducted six months after the initial evaluation and consisted of the same measures described above. 

## 3. Results

Results of gross motor function, quality of life, physical activity and human animal interactions are presented using a case model. 

### 3.1. Motor Skills

TGMD-2 locomotor scores decreased and object control scores increased. Participant 1 demonstrated a negative change in locomotor skills of −12% and a positive change of 21% in object control skills from baseline to immediate post-intervention follow-up. 

### 3.2. Quality of Life

Quality of life increased, based on self and proxy report. Based on the self-report quality of life increased 17% in the social domain, 13% in the function domain, 19% in the physical health domain, 5% in the emotional domain and there was no change in the pain domain. Based on parent proxy report, quality of life increased 42% in the social domain, 37% in the function domain, 179% in the physical domain, 103% in the emotional domain and decreased 98% in the pain domain. 

### 3.3. Physical Activity

The participant decreased sedentary behavior by 38% and increased time spent in moderate to vigorous physical activity by 300%. 

### 3.4. Adherence to Protocol 

The participant indicated at home adherence to completing the assigned activities and daily walks with the dog. However, time to complete the activities was not indicated, thus the amount of time that the participant spent each day participating in at home activities is unknown. 

### 3.5. Human Animal Bond

Improvements were seen in all aspects of the child-dog relationship over the course of therapy. The dog’s style of attachment toward the child progressed from an insecure-avoidant pattern, where the dog did not seek the proximity of the child or utilize the child as a secure base from which to explore its environment, to a secure attachment style, characterized by reunion behavior after an absence and increased exploratory activity within the testing environment. Increased sociability towards the child was also seen in the active phase of the sociability test. Time spent within 1 m of the child (proximity seeking) increased, from 12% to 62% of the 2-min period. Time spent in physical contact with the child during the two minute period increased from 0% to 25%. Proximity and contact seeking behavior remained relatively stable from baseline to post-therapy in the passive sociability condition (Proximity 4% to 5%, and contact 0% to 1%) which suggests that changes during the active phase may be more specifically tied to the dogs improved responsiveness to the child’s actions or attention getting behavior, instead of a general increase in the dog’s motivation to seek out social contact. Gesture responsiveness also improved from baseline, where the dog failed to make any choices at all during the pre-intervention assessment, to following the child’s point to the correct container 6/10 times during post intervention assessment. The dog also demonstrated a significant improvement in cooperation/task participation in general, actively selecting a container in the cooperative pointing task on all 10 of the trials (compared to zero pre-intervention).

## 4. Discussion

Improvements in aspects of motor skills, physical activity, quality of life and human animal interactions were observed, thus providing preliminary support of positive gains based on participation in an APA-AAI, using the family dog, for a child with CP. To date, the relationship between animal-assisted physical activity participation and motor skill performance has not been extensively examined. Yet, within the paucity of existing literature evidence suggests the presence of a therapy dog is associated with higher performance speeds [19] and greater adherence to instructions on locomotor (leaping, hoping, crawling) and stability (rolling over and balance beam activities) skill tasks in preschoolers with language-impairment disabilities. Gee et al. [19,20] directly examined the effect of the dog’s presence on skill performance, compared to when the dog was absent, suggesting that the inclusion of a dog in adapted physical activity interventions may serve as a strong motivator for greater participation in physical activities among children. The present study aimed to capitalize on this potential motivational mechanism through the inclusion of the family dog, by allowing for continual presence of the family dog at home and during the child’s participation in daily physical activity higher adherence was expected. Although we could not measure whether improvements in physical activity were directly associated with the intervention and the presence of the dog’s participation in physical activities the child was participating in more physical activity following the intervention, as indicated through objective physical activity monitoring. The inclusion of the family dog is novel in an APA-AAI and strong compliance, by the case presented, to the intervention supports the feasibility of this model towards improving aspects of motor skills, physical activity, quality of life and human animal interactions for a child with CP. Utilizing dogs already owned by participants may provide unique advantages that may facilitate broader access to and success of AAI programs. For example, many traditional AAI’s consist of brief interactions with therapy dogs that are owned by an independent handler [42], requiring participants to schedule one time or regular sessions to engage in the beneficial activities. This imposes time and location restrictions for participants that are ongoing. Conversely, AAI’s that facilitate joint participant-dog training allow the dyad to learn relevant skills and activities that they can continue to practice at home facilitating long-term wellness practices even after the on-site intervention sessions end or decrease in frequency. The use of family dogs may also reduce financial barriers associated with long-term therapy dog sessions or the acquisition of a specially bred and trained service dog that may cost tens of thousands of dollars upon receipt and often takes months to years to acquire [43]. Especially for AAI’s geared towards children, the ability to work with a known animal may be less intimidating and potentially safer for both the child and animal involved. Both members of the dyad, as well as the guardians, will be more familiar with each other’s body language and the dynamics of past interactions between these individuals. Research suggests that even the presence of an owned dog can have health benefits [44] and this effect is magnified when a strong bond is shared between the dog and human [45]. The use of an already bonded dog in interventions may therefore facilitate success or long term compliance in a variety of ways that are not as easily achieved with more traditional AAI approaches. Conversely, while not the case in the current study, downsides to the use of family dogs should also be considered including the possibility that some pet dogs may not be sufficiently motivated, healthy enough, or may fail to learn the behaviors required to adequately engage in intervention activities. It is also possible that regular ongoing sessions with the human AAI facilitators, while potentially costlier and more time consuming, may provide benefits beyond engaging in activities with the dog alone if and when both options are available long-term. Therefore, while the availability of a variety of AAI programs that included animal visits, specially trained service animals and family dogs will likely remain beneficial due to a diversity of participant needs across intervention and therapy contexts, the current data suggests that more research on the inclusion of family dogs in AAI is warranted. More work, in this area should ensue, including adherence of the family dog based on breed, size and temperament. Larger studies, examining this aspect of the program would be helpful. However, the current case study suggests that the inclusion of family dogs from breeds, and even dogs younger than is often typical for traditional AAI visits, may still facilitate positive therapeutic outcomes. Evidence that a one-year old Pomeranian with no prior training history can be successfully utilized in an animal-assisted adapted activity program would suggest that dogs from a much wider range of breeds, ages and sizes may be worth considering for AAI programs; minimally this question warrants further study.

Program adherence is a persistent challenge in most PA promotion programs and can be a major barrier for achieving life-long health benefits [46]. The home-based adherence of the participant in this study, strengthens the potential for an APA-AAI with the family dog to facilitate adopting life-long healthy physical activity habits [47], where the family dog may simply act as a prompt for the child to engage in healthy behaviors, like walking. More research in this area is warranted, including the types of activities which might be the most interesting and active for the participants and their family dog. Given the gains in all aspects of health inclusive of aspects of motor skill development, physical activity, quality of life and human animal interactions, the use of an APA-AAI could have far reach in respect to improving these constructs in the lives of children with CP. 

## 5. Limitations

Our current ability to generalize the findings of the present study to a broader population is limited by sample size. This family (participant) self-selected to participate in the intervention having met inclusion criteria for owning a family dog. As such, attitudes towards inclusion of the dog in the family and additional parental dog-training may have impacted behaviors and attitudes of the child in relation to canine-assisted physical activity. Inclusion of measures for child attitudes towards and perception of their interaction with their dog may combat this challenge in future studies. While only one case was reported here, observed functional and health benefits offer promising support for animal-assisted adapted activity programs with the family dog for children with CP. Decreases in some aspects of motor skills were indicated, thus authors advise replicating this novel intervention with larger more diverse samples, of children with physical disabilities and using controlled experimental designs. 

## 6. Conclusions

The case study presented here provides preliminary support for improving physical and psychosocial health through an animal-assisted adapted activity intervention with the family dog in a child with CP. Positive gains and strong adherence to daily activity protocol observed suggest facilitating activity with their family dog can promote daily PA participation and overall improvements in function and well-being. These early results warrant broader experimental examinations of the effects of animal-assisted adapted activity interventions with the family dog in children with CP and other disabilities.

## Figures and Tables

**Table 1:** Description of the physical activities completed each week during the intervention (Caveletti’s, wobble board and balance disc were substituted with walking the dog in the homework phase of the weekly intervention).

Activity	Description	Min/Repetitions
Sit-Stands	Subject’s started sitting in chair, then asked to come to a complete stand and return to sitting position.	10–15 reps
Brushing	Subjects were asked to brush their dog from front to back with each hand.	2 min each hand
Caveletti’s	Subjects walked with their dog over 4 poles, approximately 18 inches apart and 3 inches tall.	10–20 reps
Wobble board	Subjects stood on a 18” balance board with a 2” instability ball, feet shoulder width apart	3 reps for 1 min
Balance disc	Subjects stationary marched or marched in a circle on a 14” inflatable balance disc	1 min march 1 circle each direction
Fetch	Subjects played fetch with their dog, alternating hands to throw and pick up the ball	3–5 min
